# Plasticizing Diabetes Care: The Metabolic Threat of Plastic-Associated Endocrine Disruptors and Micro-/Nanoplastics in Clinical Medicine

**DOI:** 10.1007/s11892-026-01635-4

**Published:** 2026-07-13

**Authors:** Robert M. Sargis, Sirimon Reutrakul, Leonardo Trasande, Angel Nadal

**Affiliations:** 1 Division of Endocrinology, Diabetes, and Metabolism, Department of Medicine, University of Illinois Chicago, 835 S. Wolcott, Suite E625; MC 640, Chicago, IL 60612 USA; 2 Research Service, Jesse Brown Veterans Affairs Medical Center, Chicago, IL USA; 3 Chicago Center for Health and Environment, Chicago, IL USA; 4 Department of Pediatrics, New York University Grossman School of Medicine, New York, New York USA; 5 Department of Population Health, New York University Grossman School of Medicine, New York, New York USA; 6 New York University Wagner Graduate School of Public Service, New York, NY USA; 7 Instituto de Investigación en Biotecnología y Salud (IDiBE), Elche, Spain; 8 CIBER de Diabetes y Enfermedades Metabólicas Asociadas (CIBERDEM), Universidad Miguel Hernández de Elche, Elche, Spain

**Keywords:** Plastic, Diabetes, Pollution, Climate change, Bisphenol, Phthalate, Device, Pump

## Abstract

**Purpose of Review:**

Diabetes care is characterized by the widespread use of plastics. With plastic-associated endocrine-disrupting chemicals (EDCs) implicated in diabetes pathogenesis, this review examines how medical plastics relate to diabetes and related disorders and proposes interventions to improve the situation.

**Recent Findings:**

Plastic-associated EDCs and micro-/nanoplastics (MNPs) are linked to metabolic dysfunction. Medical care exposes patients to these agents; however, the precise contribution of diabetes care to these exposures and their associated adverse health effects remains poorly defined. Diabetes care is an increasingly important contributor to plastic pollution and climate change, yet inadequate systems exist to mitigate its environmental impact.

**Summary:**

Plastic-associated EDCs and MNPs remain an underappreciated metabolic health threat. Mitigating the deleterious impacts of plastics on human and planetary health requires concerted actions from manufacturers, scientists, policymakers, professional organizations, healthcare providers, and patients. Doing so has the potential to improve metabolic health and promote health equity.

## Introduction

Diabetes mellitus is a devasting metabolic disorder that currently afflicts 589 million adults globally with a staggering 853 million adults projected to have the disease by 2050 [1]. This incredible burden confers a significant toll on individuals and families as diabetes is a leading driver of non-traumatic amputations, kidney disease, and blindness as well as a potent contributor to cardiovascular disease, the leading global killer [2]. With such devastating impacts, the societal effects are unsurprisingly enormous with global diabetes-associated healthcare costs exceeding $1 trillion annually [1]. Critically, as the disease prevalence increases, the impacts of diabetes are likely to increase substantially. Thus, understanding the full array of disease drivers is critical for developing effective interventions. While dietary, lifestyle, and genetic factors clearly intersect to promote diabetes development, these factors fail to fully account for the increasing burden of disease. Moreover, interventions focused on traditional risk factors have inadequately met societal needs. As such, identifying and addressing emerging diabetes risk factors is essential, including environmental toxicants. Among such diabetogenic toxicants are chemicals found in plastics as well as the physical breakdown products of plastics, i.e. micro- and nanoplastics (MNPs). With increasing evidence linking plastic-associated chemicals and MNPs to metabolic dysfunction, plastics emerge as important diabetes risk factors. But as we consider plastics as vectors of diabetes risk, an often-neglected question arises: what are the contributions of medical care to the diabetes and plastics problem? Furthermore, if modern medicine is built on a plastic foundation, what are the consequences of that use for human health? Herein, we begin to explore these questions.

## Endocrine-Disrupting Chemicals (EDCs) and Disorders of Glucose Homeostasis

Over the last two decades, burgeoning scientific evidence has linked exposure to various toxicants to disruptions in hormonal signaling and energy metabolism, including bisphenols, flame retardants, non-essential (“toxic”) metals/metalloids (hereafter “metals” for simplicity), per-/polyfluoroalkyl substances (PFAS), pesticides, phthalates, and polychlorinated biphenyls (PCBs) among others [3–5]. The full spectrum of evidentiary links includes cellular, animal, and population-based studies with implications for obesity, metabolic dysfunction-associated steatotic liver disease (MASLD), dyslipidemia, and diabetes (reviewed extensively in [6–14]). In addition to associations with metabolic disorders that increase diabetes risk, current evidence indicates that several EDCs have the capacity to directly disrupt the key physiological pathways regulating glucose homeostasis, including both insulin secretion and insulin action, with some chemicals exerting deleterious effects on both of these parameters [14–17]. While there are classic examples of exposures to a single chemical inducing diabetes [18, 19], this is not typical. Rather human exposure is characterized by exposure to chemical mixtures, with each person exposed to a unique chemical cocktail. If some of those chemicals impair insulin secretion while others reduce insulin sensitivity, the summation of effects across those exposures may become significant enough to amplify diabetes risk, especially in the context of deleterious lifestyle factors and genetic susceptibility [12]. While EDC-mediated diabetes risk varies across individuals, the extent of population-level exposures to some diabetogenic EDCs indicate that these toxicants likely contribute to the increasing global burden of disease.

## Plastic-Associated Chemicals and Diabetes Risk

**Fig. 1:**
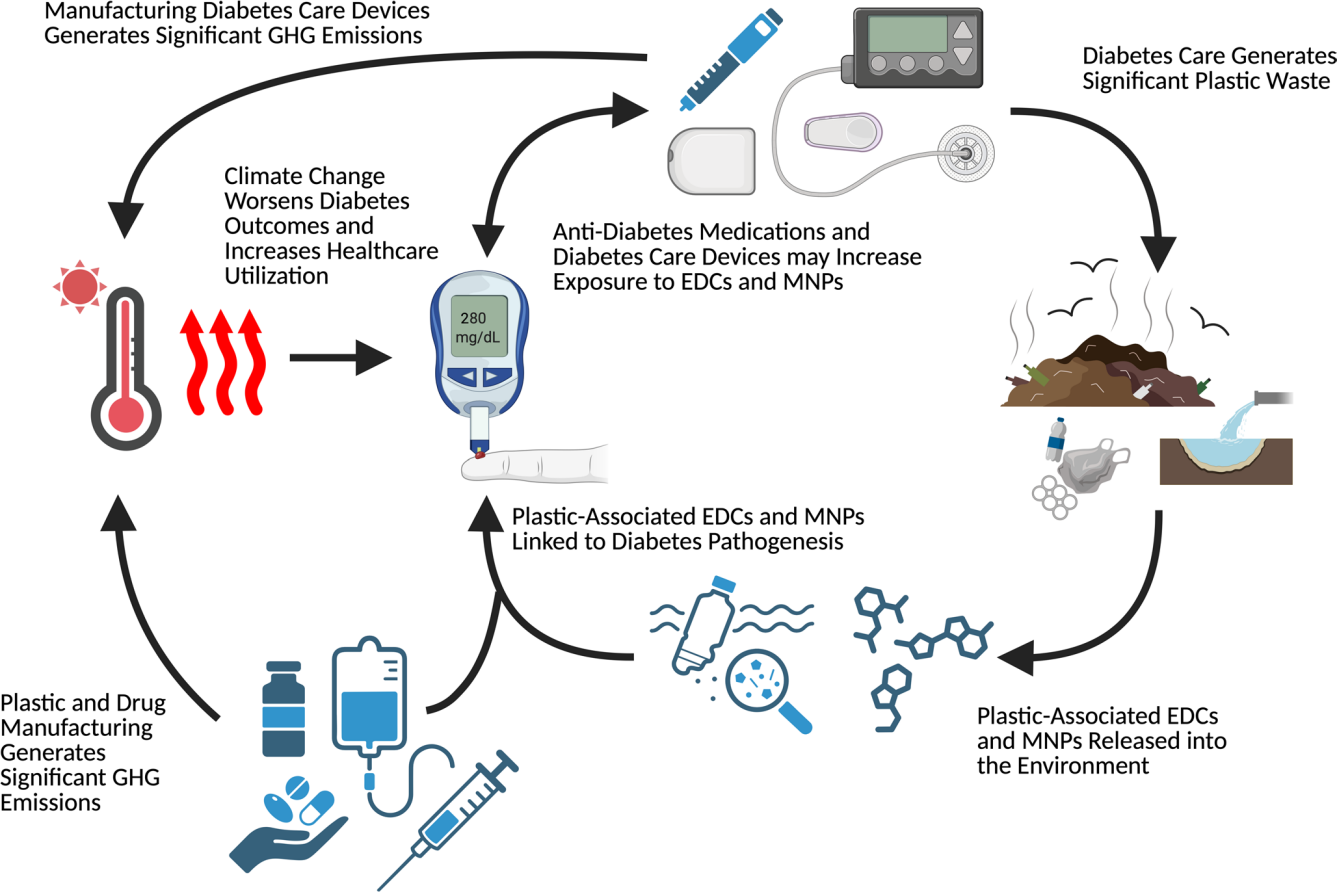
Reinforcing intersections of plastic-associated endocrine-disrupting chemicals, micro-/nanoplastics, and climate change with diabetes. EDCs, endocrine-disrupting chemicals; GHG, greenhouse gas; MNPs, micro-/nanoplastics. Created in BioRender. Sargis, R. (2026) https://BioRender.com/k0c3bgq

Plastics are complex materials that consist of both polymers and chemical additives that are used to modify the physical properties of the final product. Several of these additives exhibit one or more of the key characteristics that have been developed to define metabolism-disrupting agents, including bisphenols, phthalates, PFAS, flame retardants, UV stabilizers, toxic metals, and MNPs [9, 13, 20]. Moreover, epidemiological evidence has linked several of these toxicants to diabetes and other metabolic disorders [4–6, 8, 9, 13, 15, 16]. Importantly, the data linking plastic-associated chemicals with diabetes span the spectrum from cell-based and animal studies to population-based and clinical studies, strengthening the conclusion that toxicant exposures are linked to diabetes pathogenesis. The specific contribution, however, varies across chemicals and diabetes-related outcomes. For example, in one meta-analysis, higher versus lower urinary bisphenol A (BPA) and phthalate levels were associated with a nearly 50% increased risk of type 2 diabetes (T2D) [21], while in another study the sum of metabolites of diethylhexyl phthalate (DEHP) was associated with a greater than 2-fold increased risk of diabetes among the most highly exposed [22]. Additional meta-analyses have linked several plastic-associated EDCs with gestational diabetes (GDM), including polybrominated diphenyl ethers (PBDEs), phthalates, and PFAS [23, 24]. Importantly, our knowledge of plastic-associated diabetogenic EDCs is restricted to chemicals that have been studied, with some studied extensively and others not at all. As such, there is the potential for hidden risks. For example, antimony is a metalloid used as a catalyst in the production of polyethylene terephthalate (PET) plastic, a commonly used plastic from which antimony can migrate. While the available data are somewhat mixed, emerging evidence indicates that antimony may increase insulin resistance and disrupt glucose homeostasis [25]. More longitudinal and mechanistic studies are needed to better understand the contribution of antimony and other plastic-associated chemicals to diabetes-related outcomes; however, current evidence indicates that several plastic-associated chemicals are likely diabetes risk factors (Fig. 1). Importantly, the full impact of plastic-associated EDCs on societal diabetes burden is just beginning to emerge. While few studies have quantified the burden of diabetes attributable to EDCs in plastics, those that have examined the link suggest ~4400 incident cases of type 2 diabetes in women due to PFAS and phthalates annually, with total attributable costs of ~$400 million/year [26]. In light of the early stage of these analyses, these are likely underestimates of the plastic-mediated diabetes burden.

## Micro- and Nanoplastics (MNPs) and Metabolic Health

In addition to EDCs leaching from plastic, there are increasing concerns related to the physical breakdown products of plastics. With exposure occurring through various sources, including contaminated food, air, and water, these MNPs have been found in various bodily tissues, including brain, breast milk, kidney, liver, testis, placenta, and the vasculature among others [27–31]. Lodged into tissues, MNPs may disrupt cellular function either via direct particle-mediated effects or by serving as vehicles for delivery of plastic-associated EDCs. As such, intense research is now focused on MNP impacts on biological processes. In a highly impactful prospective study, investigators examined whether MNPs present in atheromatous plaques influenced the subsequent development of major adverse cardiovascular events (MACE) [30]. In this study, polyethylene was detected in 58.4% of plaques removed for asymptomatic carotid atheromas, while polyvinyl chloride (PVC) was detected in 12.1%. Over nearly three years of follow up, those subjects with MNPs in their atheromas were 4.53-times more likely to experience the primary MACE endpoint, raising the possibility that these particles amplify vascular risk. Such associations are increasingly supported by cell-based and animal studies linking MNPs to metabolic dysfunction. For example, polystyrene (PS) MNPs induced insulin resistance in muscle and adipose models [32, 33], while disrupting insulin signaling and raising glucose levels in zebrafish larvae [33]. In murine models, PS MNPs amplified glucose intolerance while reducing insulin sensitivity and energy expenditure, effects potentially attributable to mitochondrial dysfunction [34], a phenomenon also observed in muscle cells [32]. PS MNPs also altered lipid handling in adipose tissue and liver [35]. In addition, they increased glucose intolerance, insulin resistance, and dyslipidemia, with MNPs amplifying a subset of effects induced by a high-fat diet, including adipocyte hypertrophy [36–38]. Interestingly, inhaled PS MNPs reduced body weight, but despite this reduction, exposed mice similarly exhibited fasting hyperglycemia, glucose intolerance, and insulin resistance with associated hepatic injury [39]. In addition to mitochondrial dysfunction and altered insulin signaling, mechanisms linking MNPs to altered energy metabolism may include changes in the gut microbiome [40, 41] and enhanced immune activation, including in the hypothalamus and liver [37, 42]. In a pig model, exposure to PET MNPs also disrupted metabolic parameters [43]. Importantly, in addition to effects of the particles themselves, MNPs may amplify the adverse effects of EDCs on metabolic tissues [44–46]. Collectively, these data are intriguing; however, the science of interrogating MNP effects on human health is rapidly evolving with many studies requiring validation to confirm the presence and biological impact of MNPs in human tissues [47, 48]. Despite some lingering uncertainties, however, the prevalence of plastic in our lives and the available scientific evidence suggest that MNPs are novel metabolic disease risk factors.

## Healthcare as a Source of Exposure to Diabetogenic EDCs

It is becoming abundantly clear that our vast and increasing use of plastics carries with it health risks that have heretofore been largely ignored. This recognition is acutely jarring in the context of modern medicine, which is a realm dominated by plastic use. The question is to what extent plastic use in healthcare exposes patients to plastic-associated EDCs and MNPs and how those exposures may impose risks or limit benefits of plastic-dependent medical care. At present, various EDCs are known to be released from medical devices and supplies, including bisphenols, parabens, PFAS, phthalates, and triclosan among others. In one analysis of common medical supplies, syringes released phthalates, bisphenols, and parabens; microcapillary blood tubes released parabens; venous catheters released bisphenols, parabens, and phthalates; and parenterally administered fluids carried parabens, phthalates, and other potential EDCs [49]. The prominence of phthalates in this study is not surprising as this class of chemicals accounts for 30–40% of the weight of medical grade plastics [50, 51]. Importantly, phthalate leaching is enhanced in lipophilic solutions such as blood and parenteral nutrition [52]. Among the critically ill, potential phthalate exposure sources are myriad, including intravenous bags, extension lines, and infusion sets as well as blood bags, endotracheal tubes, and cardiopulmonary bypass machines [50, 52–54]. In one French study, 75% of hospitalized pregnant women were exposed to at least one phthalate-containing medical device [51]. Patient exposure to BPA is likely most significant during dialysis [55] but can also occur from many other sources [56]. Indeed, urinary BPA levels are associated with the number of medical devices used in a patient’s care [57]. Parabens are EDCs with antimicrobial properties that are incorporated into ultrasound gels and intravenous solutions [49, 58], with some health systems using parabens in heparin lock solutions to prevent catheter-associated bloodstream infections, for which they may have clinical utility [59]. PFAS are present in medical equipment and devices, including in medical tubing and sutures [60].

The Neonatal Intensive Care Unit (NICU) represents a unique clinical scenario in which exposures to several EDCs occur at both high levels and during a critical developmental window, including phthalates, BPA, and parabens. In an analysis of 52 NICU items, 60% of equipment contained BPA, while 80% contained parabens; moreover, extracts from these devices exhibited endocrine activity, with 25% and 10% exhibiting estrogenic activity and anti-androgenic activity, respectively [61]. First identified in 2004 [62], it has now long been suggested that phthalate exposures in the NICU are likely to exceed safe levels [63]. Interestingly, higher BPA levels are seen in NICU patients exposed to phthalate-containing medical products, indicating important co-exposures in this setting [64]. In a more recent analysis of hospitalized very low birth weight infants, estimated daily intake of EDCs frequently exceeded tolerable daily intake, including BPA (100%), DEHP (75%), sum of benzylbutyl phthalates (BBPs) (90%), and propylparaben (50%) in the first week, while estimated daily intake remained above the tolerable daily intake in the fifth week for BPA (100%), DEHP (75%), BBPs (75%), and propylparaben (25%). Earlier age of birth and late-onset septicemia resulted in higher exposures with levels considered sufficient to increase the risk of adverse health effects [65]. Such risks are supported by evidence that phthalates are associated with broncho-pulmonary dysplasia, necrotizing enterocolitis, parenteral nutrition-associated cholestasis, and neurodevelopmental disorders [66]. While causal mechanisms are likely complex with potential confounding from underlying medical conditions, it is intriguing that switching from phthalate-containing medical devices may reduce the risk of conditions such as total parenteral nutrition-associated cholestasis [67].

Recognizing the risks of exposure to EDCs through medical equipment, in 2021 the European Union restricted the use of DEHP, one of the most used plasticizers, in medical devices. In a recent analysis in Belgium, phthalate metabolites were detected in 90–100% of neonates [68]. While DEHP was lower than in historical comparators, higher levels of other plasticizers were detected, with some levels substantially high. Indeed, phthalate levels exceeded animal-based no effect levels in 10% of neonates, while 29% of premature neonates had at least one phthalate measurement above these thresholds. Approximately, 57% of neonates had one exposure at a level that may exert deleterious effects on long-term development. Respiratory support and the administration of blood products were associated with higher phthalate levels [68]. Thus, while concerns about EDCs in medical devices have received greater attention with consequential regulatory action, mitigation challenges persist.

Importantly, exposure to plastic-associated chemicals among patients is not restricted to hospitalized patients or the use of medical equipment. Several medications also contain phthalates [4]. These chemicals are used as excipients in medications to control their release in the gastrointestinal (GI) tract, and various drugs used to treat GI illnesses contain phthalates [69]. Moreover, use of such agents is linked to higher urinary phthalate levels in NHANES analyses [70]. But phthalate use in medications goes beyond GI drugs; analyses in the US, Canada, and Denmark revealed phthalates in a variety of prescription and over-the-counter medications [71, 72]. In NHANES analyses, use of several medications was linked to higher phthalate levels [73], while a Danish study revealed dibutyl phthalate concentrations exceeded recommended levels in 50–90% of patients by as much as 6-fold [74]. Importantly, these phthalate exposures may be associated with hormonal disruptions [75], and a registry analysis from Denmark suggested that maternal and childhood exposure to phthalates via medications was associated with an increased risk of osteosarcoma and lymphoma [76]. In addition to prescribed medications, dietary supplements may also be a phthalate exposure source [77]. Beyond phthalates, medications may contain other EDCs, including parabens, chemicals Generally Recognized As Safe (GRAS) by the FDA and as such exempt from tolerance requirements [78]. Data indicate that levels of various parabens may be increased by exposure to paraben-containing medications [78, 79]. In addition to potential impacts mediated by their endocrine-disrupting properties [4], the antimicrobial properties of parabens may implicate them in metabolic dysfunction through alterations in the gut microbiome [13]. Finally, some medications and chemicals used in pharmaceutical manufacturing are PFAS, raising questions about the sustainable use of such agents [80].

Data on MNP contact via medical care are just emerging, with most studies simulating clinical scenarios to quantify potential MNP exposures that may enter the human circulation. In one such simulation examining MNPs emanating from crystalloid intravenous fluids, each liter of fluid generated approximately 7500 polypropylene particles ranging in size from 1 to 62 μm [81]. Other studies have detected various MNP types in saline infusion set-ups, including polypropylene (PP), PVC, PS, polymethyl methacrylate, PET, polyamide, and polyethylene (PE) [82–87], as well as in blood collection needles [88]. Importantly, PVC MNP concentrations in intravenous fluids increased at higher temperatures and with longer infusion times [89]. In an analysis of parenteral nutrition, MNP concentrations (most abundantly those of PET origin) were higher in lipid emulsions than crystalloids [90]. Notably, built-in filtration membranes can lower MNP concentrations in the final infusate; however, they do not completely retain MNPs and may release their own particles [84, 90, 91]. Importantly, studies demonstrating the widespread presence of PE, PP, and PS MNPs in infusions have also shown that PP and PE MNPs were positively associated with phthalate levels [91–93], indicating links between MNPs and plastic-associated chemicals. To date, most studies of MNPs in medical devices and equipment are restricted to simulations and have not quantified circulating MNPs in humans before and after such infusions. Ultimately, understanding true in vivo exposures will be critical for quantifying risk.

## Diabetes 2026: Revelatory Change, at a Potential Cost

Diabetes care has been transformed over the last two decades with the rising tide of disease being met by a strengthening wall of therapeutics with proven clinical benefits. Pharmacologically, our anti-diabetes armamentarium now includes agents that not only decrease blood sugars, but that also reduce microvascular complications [94–97], address common comorbidities and predisposing factors (e.g., obesity, MASLD, obstructive sleep apnea, hypertension) [98–103], reduce prediabetes-to-T2D progression [98, 104], and improve cardiovascular outcomes [105–109]. Many of these benefits have been driven by the biologics revolution that has provided peptide- and protein-based drugs that address underlying metabolic defects common in diabetes; these currently include GLP-1RAs and GIP/GLP-1R dual agonists, with a host of additional agents in the pipeline targeting additional receptors such as those for glucagon, amylin, and others [110–112]. Importantly, while there are oral formulations of semaglutide [113, 114] and small molecule GLP-1R agonists in development [115], the vast majority of these revolutionary agents are administered subcutaneously mostly using single-use disposable plastic devices. Such devices are also widely used for the administration of insulin, a medication that remains essential in all cases of type 1 diabetes (T1D) and in many cases of T2D. Importantly, these devices have advantages over traditional vial and syringe administration [116], and as such, are recommended by the American Diabetes Association (ADA) as the preferred insulin delivery method for most patients [2]. Importantly, such administration aids are not restricted to diabetes care, rather similar devices are widely used for the administration of biologic agents across medicine.

With plastic pens central to pharmacology, there is potential for diabetes care to contribute substantially to global plastic pollution, especially since few if any programs are available to reuse or recycle these devices. Novo Nordisk introduced its ReMed™ program allowing patients to return their injection pens for recycling [117]. However, this program is not universally available, with the US notably excluded as of this writing. While pens that can be repeatedly reused would be ideal, devices should at least be able to delivery multiple medication doses to decrease plastic waste when there is a long-term therapeutic need. This is why it has been perplexing that Mounjaro® and Zepbound® pens in the US are single-use, while multidose alternatives have been available in other countries. It seems that the drugs’ manufacturer is finally launching multi-dose pens; however, this has taken 5 years, and it remains unclear whether single-dose devices will be fully eliminated [118]. Unnecessarily generating plastic waste when less wasteful alternatives are available is poor environmental stewardship. With the explosion in plastic medication delivery device utilization, there is a mandate for better practices. Since recycling generates outputs of lower quality than the initial material, recycling programs should be the bare minimum demanded of manufacturers for handling their products’ life-cycle. Preferred would be devices that allow for repeated reuse over extended periods of time.

In addition to the pharmacological revolution, rapid advances in medical device technologies have transformed diabetes care, especially for those needing insulin. Continuous glucose monitors (CGMs) now provide near real-time assessment of glucose levels with the capacity to provide patients with early warning of imminent hypo- and hyperglycemia while also empowering advanced insulin pumps [automated insulin delivery (AID) systems] that assist patients in self-care by dynamically responding to insulin needs. The benefits of these devices are enormous, improving clinical outcomes and quality of life (reviewed extensively in [2]). As such, CGM utilization has grown dramatically, with the ADA now recommending CGM use for children, adolescents, and adults with diabetes who are on insulin or other therapies that can induce hypoglycemia as well as at times when CGM can aid disease management [2]. Furthermore, for those on insulin, the recommendation is for CGM use as near as to daily as possible [2]. With regard to AID systems, their clear benefits has prompted the ADA to formalize their centrality to insulin-based diabetes care, with the current Standards of Care noting the preferred status of AID-CGM systems for insulin delivery in people with T1D, adults with T2D, children and adolescents with T2D, and those with other forms of insulin-deficient diabetes [2]. These guidelines coupled with recent FDA clearance of multiple AID systems for use in patients with T2D will undoubtedly increase the use of AID systems [119–121]. With this increased utilization, there will assuredly be a concomitant increase in plastic waste generation.

While these agents and devices have had transformational benefits for patients, their use raises concerns about the impact of modern diabetes care on the exposure of patients to plastic-associated chemicals and MNPs as well as on the environmental costs of plastic waste generation. With regard to potential impacts on the patient, several questions arise. First, do these devices increase exposure to plastic-associated EDCs and/or MNPs? Second, if so, do these exposures dampen therapeutic efficacy or contribute to adverse effects? Third, could alternative devices offer similar benefits without incurring potential adverse impacts of plastic-associated EDC and MNP exposure? Unfortunately, the answers to these questions are largely unknown. In one case-control study, subjects with diabetes on insulin had higher blood MNP levels compared to healthy controls, with MNP levels correlated with HbA1c; however, the number of daily injections was not associated with MNP levels, and the results were likely confounded by substantial differences between the comparison groups [122]. Studies are needed to better understand how current treatments impact plastic-associated exposures and diabetes-related outcomes to better understand potential risks to patients.

In addition to potential patient exposures, there is the broader impact of plastic waste resulting from the disposal of these devices and the consequential dissemination of EDCs and MNPs into the wider environment. Indeed, the threat of plastic pollution to human and planetary health is enormous [123], with each stage of the plastics lifecycle a danger, from feedstock extraction to product manufacturing and use to recycling and environmental disposal [124]. While the contribution of medical devices and equipment to total global plastic pollution is difficult to find, their contribution is likely substantial. In a recent study of waste generated by individuals with insulin-dependent T1D and T2D, investigators quantified diabetes-related waste over 30 days, including insulin cartridges, bottles, inhalers, needles, syringes, glucose test strips and meters, lancets, alcohol wipes, insulin pumps, and CGM sensors [125]. Not included were other important waste products, including glucagon products, tape, skin care products, packaging, instructions, batteries, and insulin infusion sets and cartridges. Based on their analysis, extrapolated mean annual waste generated by individuals on insulin pumps was estimated to be 14.95 kg/year (SD: 6.19 kg/year), while those on multiple daily injections of insulin were 16.81 kg/year (SD: 18.96 kg/year). With the US alone having approximately 8.4 million people living with diabetes on insulin, including approximately 2 million with T1D, the potential contribution of diabetes to plastic and other medical waste is enormous [125]. Moreover, with increasing use of disposal insulin pumps and CGMs coupled with expanded use of those devices in individuals with T2D, the impact of diabetes care on plastic waste is likely to skyrocket.

## Perverse Incentives from Disordered Healthcare: The Case of Insulin Pumps

Insulin pumps come in essentially two varieties, tubed pumps and tubeless or patch pumps. The diversity of these systems gives patients substantial choice for how best to manage their diabetes. Indeed, the current ADA guidelines state that AID choice should be made based on the patient’s circumstances, preferences, and needs [2]. But that choice has potentially profound environmental impacts. Even though AID systems perform very similar functions, the simple presence or absence of a tube can markedly change health insurance coverage. When covered by insurance, most tubed pumps are considered durable medical equipment and most often covered by a patient’s medical benefit, while tubeless/patch pumps are disposal and covered under the pharmacy benefit. The impacts of this distinction are potentially significant. Due to deductibles and coinsurance, tubed pumps may incur higher upfront costs; furthermore, there are often delays in receiving approval for durable medical equipment, and once procured, the commitment to that pump is expected to last several years. In contrast, tubeless/patch pumps are covered by the pharmacy benefit and often have lower, predictable monthly copays and can be procured more easily without a long-term commitment to the same device. From an environmental perspective, this is significant since tubeless pumps are disposable, and as such, generate more plastic and electronic waste (e-waste). To our knowledge, the impact of medical versus pharmacy benefit coverage on insulin pump choice has not been explored, nor have the environmental impacts of that choice. A recent cost-effectiveness analysis suggested that a tubeless AID device was cost-effective in T1D when compared to standard of care; however, this finding didn’t address the comparative cost-effectiveness of modern tubed vs. tubeless/patch AID systems [126]. Moreover, environmental costs have never been considered in cost-effectiveness analyses. Addressing these knowledge deficits will be critical for empowering patients with the information they need to make decisions that conform to both their clinical needs as well as their values. The recent launch of a recycling program for OmniPod’s tubeless pump may assist in those value assessments. However, once again this program was instituted many years after their pumps entered the marketplace, and it remains to be seen how effective this program will be in terms of waste diversion and product reuse.

## Beyond Chemicals: Plastics, Climate Change, and Diabetes

The greenhouse gas (GHG) emissions driving anthropogenic climate change arise largely from the burning of fossil fuels. Those same fossil fuels are used to produce plastics, creating a clear link between climate change and plastics [123]. This is important because people living with diabetes may be uniquely sensitive to climate change. Our recent analysis of the intersection between diabetes and climactic factors such as the extreme temperatures and natural disasters suggested that climate change was likely associated with worsening glycemic control, greater healthcare utilization, and increased mortality [127]. Further work is needed to clarify the impacts of climate change on diabetes-associated disease complications and non-glycemic metabolism as well as effects in specific subsets of patients who are expected to be especially vulnerable to the impacts of extreme temperatures and natural disasters, including those with T1D, GDM, and pediatric diabetes. Furthermore, there is a need for more data from low- and middle-income countries, many of which are predicted to experience greater impacts from climate change. Despite these data gaps, current evidence suggests that our reliance on plastics in the care of individuals with diabetes may indirectly expose those individuals to secondary metabolic and healthcare access stresses stemming from fossil fuel-driven climate change.

## Ethical Demands of Plasticized Medicine and Implications for Health Justice

The ethical principles of beneficence, non-maleficence, justice, and autonomy guide the practice of medicine. These principles are foundational to informed consent and axioms of medical care such as “first do no harm.” EDCs in medications and medical devices challenge the provision of ethically just healthcare because their presence is rarely known by providers much less communicated to patients [128]. Thus, risks are incompletely informed. While the benefits of plastics-empowered modern healthcare may far outweigh the risks of plastic-associated chemical and MNP exposures, we often make that determination without knowledge of the exposures or their associated health impacts. Moreover, while medications and medical devices likely differ in their EDC composition and consequential impact on patients, providers rarely know of or have access to better alternatives. The solutions to this knowledge deficit are not to abandon modern medicine. Rather we need better knowledge of exposures, impacts, and alternatives; collectively such knowledge will not only ensure the provision of ethically sound care, but it will also allow us to optimize therapeutic efficacy and reduce unwanted side effects.

The ethics of diabetes care is also intertwined with the fact that diabetes is characterized by substantial health disparities, with the condition disproportionately burdening low-income communities and communities of color [129]. Indeed, diabetes risk is profoundly influenced by the social and structural determinants of health (SDOH). While many SDOH factors are frequently discussed in the context of diabetes, the contribution of environmental exposures to disease disparities are incompletely appreciated despite the fact that the same communities disproportionately burdened by diabetes also have higher exposures to a variety of metabolism-disrupting EDCs [130–132]. While these exposures stem from a variety of sources, the contribution of medical care to those exposures may be substantial in some individuals, particularly among those receiving treatment with many medications and devices. Thus, individuals with a higher burden of disease are likely to have a higher burden of exposures, potentially exacerbating their clinical state.

Importantly, the burden of healthcare-associated exposures may be influenced by the availability of treatment options, including those related to diabetes care. Indeed, access to and use of insulin pumps is more common among those of higher socioeconomic status [133]. Given the substantial benefits of these devices, improving device dissemination across all individuals will be an important step in promoting diabetes health equity [134–136]. What such moves toward greater equity in diabetes care may mean regarding EDC and MNP exposures requires further study, but it also reinforces the need to ensure that modern diabetes care does not carry with it a cryptic risk of exposure to chemicals with adverse health effects that may amplify other health disparities.

## The Path Forward

As our understanding of plastic-associated health risks matures, several fundamental questions arise, including: 1) how can our reliance on plastics be reduced; and 2) how can the unintended adverse health and environmental impacts of plastic-associated chemicals and MNPs be mitigated? In considering these questions, it becomes clear that effectively reducing the threat of diabetogenic plastic-associated EDCs and MNPs arising from modern diabetes care will require action from multiple stakeholders, including pharmaceutical and device manufacturers, the health insurance industry, professional organizations, policymakers, the research community, care organizations, clinicians, as well as patients and their advocates (Table 1). Each of these stakeholders should be aligned in common cause to reduce the need for plastics and, when that is not possible, ensure that plastics are as safe as possible for both human health and the broader environment. In so doing, we will address a largely neglected driver of diabetes vulnerability and the untold havoc it wreaks on people living with or at risk of developing the disease.

**Table 1:** Stakeholder-based interventions to understand and address plastic-associated endocrine-disrupting chemicals and micro-/nanoplastics linked to diabetes and other related metabolic disorders

Primary Stakeholder	Action
Pharmaceutical and Device Manufacturers	Develop drug delivery devices that are resusable; when not reusable, devices should be recyclable. Final products should minimize plastic waste.
	Test all products for endocrine disrupting activity before releasing them into commerce.
	Disclose endocrine-disrupting chemical (EDC) content of pharmaceutical products and medical devices.
	Develop and implement drug preparations and devices that minimize exposure to EDCs and micro-/nanoplastics (MNPs).
	Develop and market products free of EDCs and MNPs.
	Adopt circular economy principles, including ownership over the full life-cycle of all products.
	Develop oral alternatives to injectable agents to minimize reliance on plastics.
	Develop longer-acting drug formulations that reduce the need for frequent injections.
	Develop devices that can be used for extended periods of time (e.g., extended wear infusion sets and continuous glucose monitors).
	Offer, promote, and lead recycling programs for devices and diabetes care supplies.
Research Community	Characterize EDC and MNP exposures arising from diabetes care, including those from medications and devices.
	Determine the health effects of EDCs and MNPs encountered during the provision of medical care, including exposures occuring during sensitive windows of development.
	Determine the consequences of plastic utilization and pollution on health.
	Investigate the benefits of alternative products designed to minimize EDC and MNP exposures.
	Develop clinical tests to quantify EDC and MNP exposures in patients.
	Clearly integrate environmental exposures and climate change into studies of and efforts to address the social and structural determinants of health.
Health Insurance Industry	Develop economic systems to incentivize manufacturers to minimize plastic waste, EDC and MNP exposures, and greenhouse gas emissions.
	Ensure insurance coverage systems favor approaches that minimize adverse environmental impacts while ensuring the broadest access to cutting-edge therapies that improve health outcomes and quality of life while minimizing adverse health effects.
	Ensure that insurance coverage of diabetes management does not adversely impact communities disproportionately burdened by diabetes and other environmental health threats.
	Cover the costs of clinical tests to assess exposure to EDCs and MNPs.
Professional Organizations	Advocate for government funding for scientific studies to identify and address health risks stemming from EDC and MNP exposures arising from medical care or other sources.
	Advocate for public policies that address the environmental drivers of diabetes risk and disparities.
	Incorporate environmental health into clincial practice guidelines.
	Advocate for incorporation of environmental health into medical education.
	Demand development and insurance coverage of clinical tests to assess exposure to EDCs and MNPs.
	Integrate environmental health sessions into scientific meetings.
	Inccorporate environmental health in board certification requriements.
	Develop guidance on the ethical use of plastic products in clinical care that aligns with such core prinicples as beneficience, non-maleficence, justice, and autonomy among others.
Policymakers	Fund scientific studies to examine EDC and MNP exposures and their health effects arising from medical care.
	Fund scientific and implementation studies that explore alternatives to plastic use by the healthcare industry.
	Incentivize systems in which medical devices and products can be reused.
	Mandate and enforce regulations that all non-reusable plastic and e-waste be recycled.
	Require manufacturers to assume responsibility for the full life-cycle of their products.
	Develop regulations that ensure similar coverage for tubed and tubeless insulin pumps while also developing incentive structures that favor choices that generate less waste.
	Support a global plastics treaty that aggressively addresses the environmental and health threats of plastics.
Care Organizations	Implement practices that reuse medical products.
	Reduce plastic waste production and greenhouse gas emissions.
	Demand disclosure of EDC content and potential MNP exposures from drug and device manufacturers.
	Choose devices and equipment that minimize patient exposure to EDCs and MNPs.
	Educate health care practitioners about the risks of EDCs and MNPs.
Clinicians	Become knowledgeable about the risks of plastic pollution and climate change to human health.
	Educate patients about the health threats of plastic pollution and climate change.
	Advocate for policies and practices that limit environmental health risks.
	Develop educational systems that empower patients to choose care plans that mimize EDC and MNP exposures as well as environmental contamination.
	Implement systems that reduce the environmental impacts of clinical care in healthcare systems.
Patients and Patient Advocacy Organizations	Consider environmental impacts when choosing approaches to diabetes self-care.
	Take advantage of programs to reuse and recycle diabetes care products.
	Demand products that minimize unnecessary exposures to EDCs and MNPs.
	Hold elected officials accountable for developing, implementing, and enforcing policies that protect human and environmental health.
	Demand the development and implementation of clinical tests to quantify exposures to EDCs and MNPs.

## Conclusions

Plastic-associated chemicals and MNPs from medications and medical devices are underappreciated threats to metabolic health. While further work is needed to clarify their full impact on people living with and at risk of diabetes, current evidence makes clear that action is needed. Healthcare providers must demand that action and ensure that stakeholders across society are aligned in ensuring that the patients for whom we care are provided optimal access to care that maximizes clinical benefits while minimizing unnecessary threats from plastics.

## Key References

- Nadal A, Fuentes E, Muncke J, Sargis RM. Microplastics, nanoplastics, and plastic chemicals: applying the key characteristics of metabolism disrupting agents shows reason for concern. Environmental Endocrinology. 2026;2(1):wkag001.
  - ○ This article uses a key characteristics framework to compile evidence linking plastic-associated chemicals and micro-/nanoplastics with diabetes and other metabolic disorders.
- Trasande L, Krithivasan R, Park K, Obsekov V, Belliveau M. Chemicals Used in Plastic Materials: An Estimate of the Attributable Disease Burden and Costs in the United States. J Endocr Soc. 2024;8(2):bvad163.
  - ○ This recent analysis begins to quantify the contribution of plastic-associated chemicals with diabetes disease burden and associated healthcare costs.
- Marfella R, Prattichizzo F, Sardu C, Fulgenzi G, Graciotti L, Spadoni T, et al. Microplastics and Nanoplastics in Atheromas and Cardiovascular Events. N Engl J Med. 2024;390(10):900-10.
  - ○ This groundbreaking study linked the presence of micro-/nanoplastics in atherosclerotic plaques with a markedly increased risk of subsequent major adverse cardiovascular events.
- Panneel L, Cleys P, Poma G, Ait Bamai Y, Jorens PG, Covaci A, et al. Ongoing exposure to endocrine disrupting phthalates and alternative plasticizers in neonatal intensive care unit patients. Environ Int. 2024;186:108605.
  - ○ This recent analysis highlights continued exposure to endocrine-disrupting chemicals in the neonatal intensive care unit while emphasizing the likelihood that some of these exposures exceed levels considered to be safe.
- Tian T, Ho CN, Ayers AT, Aaron RE, Klonoff DC, Ahn DT, et al. Quantifying Environmental Waste From Diabetes Devices in the U.S. Diabetes Care. 2025;48(7):1198-203.
  - ○ This study is among the first to quantify the tremendous burden of medical waste generated from the care of patients living with diabetes.

## Data Availability

No datasets were generated or analysed during the current study.
